# Longitudinal Evidence for Age- and Sex-Invariant Dimensions of Internalizing Symptoms from Childhood through Adolescence: Self-Worth, Social Anxiety, and Withdrawal

**DOI:** 10.1007/s10802-026-01474-7

**Published:** 2026-07-04

**Authors:** Arash Mehrkesh, Misaki N. Natsuaki, Tuppett M. Yates, Jenae M. Neiderhiser, Daniel S. Shaw, Jody M. Ganiban, Leslie D. Leve

**Affiliations:** 1 Department of Psychology, University of California, 900 University Avenue, Riverside, CA 92521 USA; 2 Department of Psychology, Penn State University, University Park, PA USA; 3 Department of Psychology, University of Pittsburgh, Pittsburgh, PA USA; 4 Department of Psychological and Brain Sciences, George Washington University, Washington, DC USA; 5 Department of Counseling Psychology and Human Services, University of Oregon, Eugene, OR USA

**Keywords:** Measurement invariance, Internalizing symptoms, Longitudinal, CBCL, Exploratory structural equation modeling

## Abstract

Internalizing problems rise during adolescence, yet the stable underlying dimensions remain unclear. Using two longitudinal samples (Sample A: *N = 488*, 43% girls at birth, adopted children, ages 7–16; Sample B: *N = 222*, 50% girls at birth, biological children from low-income families, ages 7–17), we examined dimensions of internalizing symptoms (excluding somatic complaints) on the Child Behavior Checklist using exploratory structural equation modeling (ESEM) and confirmatory factor analyses. In Sample A, three dimensions emerged: low self-worth/depression (LSW/Dep), social/general anxiety (S/GA), and depression/withdrawal (Dep/With). In Sample B, S/GA and Dep/With combined into the social anxiety/withdrawal (SA/With) factor, alongside LSW. Both solutions were largely stable across age and sex. Factor trajectory analyses revealed that levels of all dimensions increased from childhood to early adolescence, for both sexes. Trajectories subsequently diverged: LSW/Dep became more pronounced in girls only, S/GA increased in girls and declined in boys, and Dep/With showed the steepest increase for both sexes. Dimensions showed strong homotypic but weak heterotypic continuity. Findings highlight distinct developmental and sex-specific pathways of internalizing vulnerability detectable in middle childhood, supporting earlier and more targeted identification and intervention.

Internalizing problems, including depressive, anxious, and somatic symptoms, constitute one of the main categories of mental health problems that begin in early childhood and escalate significantly during adolescence, particularly among girls, who are about twice as likely to meet clinical thresholds for depression and anxiety during adolescence (Costello et al., 2011; Hilt & Nolen-Hoeksema, 2009; Keyes & Platt, 2024; Zahn-Waxler et al., 2008). The severity and prevalence of internalizing symptoms have intensified over the past three decades (Blomqvist et al., 2019), especially following the onset of the COVID-19 pandemic (Deng et al., 2023). While a large body of research has focused on the development of internalizing symptoms, most studies either aggregate symptoms into total scores or classify them under broad psychiatric diagnoses such as depression or anxiety (Chentsova-Dutton & Ryder, 2025). However, growing evidence suggests that these diagnostic categories—particularly depression, and to a lesser extent anxiety—may lack developmental sensitivity. These categories are often heterogeneous, overlapping, and psychometrically unstable, especially among youth (Beesdo et al., 2009; Fried & Nesse, 2015a, b; Price et al., 2013; Watson et al., 2022; Weisz et al., 2023). Therefore, this study had two primary aims: (a) to uncover the distinct age- and sex-invariant dimensions of internalizing symptoms between ages 7 and 17 by testing the measurement invariance (MI) of their factor structure, and (b) to compare the developmental trajectories of these dimensions across age and sex, including tests of their longitudinal continuity and cross-lagged associations.

## Internalizing Symptoms Across Development: Patterns and Methodological Concerns

Internalizing symptoms encompass a wide range of affective and emotional manifestations that exhibit age-specific patterns of prevalence and profile. As a general trend, levels of internalizing symptoms are likely to increase from childhood to adolescence (Klinge et al., 2025; Steinsbekk et al., 2022); however, this developmental trajectory varies by symptom type and sex. In particular, generalized anxiety and social anxiety tend to increase or remain stably elevated during mid- to late adolescence for girls, and to a lesser extent for boys (Nelemans et al., 2014; Steinsbekk et al., 2022; Tng et al., 2025), while separation anxiety and panic symptoms decline during adolescence, with steeper declines in boys (Nelemans et al., 2014; Voltas et al., 2016). Depressive symptoms typically emerge or intensify during the transition to adolescence, particularly among girls, with a discontinuity around age 12 when symptoms begin to rise sharply (Cohen et al., 2017; Dekker et al., 2007). However, these observed trajectories may not reflect true symptom resolution or emergence; a decline in one symptom form may represent a transformation into more developmentally salient expressions, while an apparent absence may reflect limited developmental salience rather than an absence of psychopathology (Beesdo et al., 2009; Blok et al., 2022; Weems, 2008). Accurately tracing these patterns therefore requires that the measurement tools used to assess them function equivalently across age and sex, a central but frequently overlooked methodological step.

From a psychometric perspective, comparing symptoms across development without establishing MI risks confounding genuine developmental change with measurement artifacts. MI techniques can identify which symptoms load onto the same psychological dimensions across groups, and which symptoms are more readily expressed at lower levels of psychopathology. Although evidence is mixed across developmental windows, measures, and informants, some studies suggest that certain internalizing symptoms shift in relevance across age. Based on parent-report instruments, for instance, unhappiness and worry are more frequently endorsed in adolescence than in childhood even when underlying distress remains stable, as shown on the Strengths and Difficulties Questionnaire (SDQ) (Riglin et al., 2024) and the Social Behavior Questionnaire (SBQ) (Murray et al., 2019). Based on child and adolescent self-reports, symptoms from the Children’s Depression Inventory (CDI), such as hopelessness and social withdrawal, are more strongly associated with depression at later ages (Van Beek et al., 2012), and anxiety may appear as fear and behavioral inhibition in early childhood but later manifest as cognitively elaborated forms, such as social anxiety in adolescence (Hirshfeld-Becker et al., 2008; Mathyssek et al., 2013).

The same uncertainty applies when comparing symptoms across groups, and particularly across sex, based on evidence of distinct developmental trajectories during adolescence. Among adolescent self-report measures, sex-related non-invariance has been documented on the Beck Depression Inventory-II (Keller et al., 2020; Wu & Huang, 2014) and the Youth Self-Report (Fonseca-Pedrero et al., 2010), with girls more readily endorsing tearfulness and diminished self-worth and boys more readily endorsing pessimism and concentration difficulties at equivalent symptom levels. Among parent-report instruments, mother-reported data from the Child Behavior Checklist similarly revealed differential item endorsement by sex, with “cries a lot,” “shy/timid,” and “enjoys little” showing bias in a large clinical sample (van der Sluis et al., 2017). Critically, whether sex-related non-invariance emerges, and for which items, varies considerably across samples, instruments and informants; MI may hold fully, partially, or not at all. This uncertainty motivates formal testing prior to any group-level comparisons, so that any observed differences in latent means can be attributed to genuine psychological variation rather than measurement artifacts.

### Evidence for Distinct Dimensions of Internalizing Symptoms, and Their Trajectories

Our understanding of mental health problems has increasingly shifted away from a categorical view of psychopathology, such as that used originally in the DSM (American Psychiatric Association, 2022), toward a more dimensional one, supported by empirical and clinical perspectives. For instance, in the HiTOP (Hierarchical Taxonomy of Psychopathology) framework, statistical analyses support the existence of well-replicated spectra (Kotov et al., 2017). Specifically, within the internalizing symptoms cluster, two distinct yet correlated subfactors—distress and fear—are frequently extracted; together, they form the broader internalizing factor (Krueger & Eaton, 2015). The distress factor includes major depression, dysthymia, and generalized anxiety, whereas the fear factor is mainly characterized by context-specific forms of psychopathology, such as specific phobia, social anxiety, and agoraphobia (Watson et al., 2022).

Clinical and theoretical perspectives offer related, yet distinct, formulations of dimensions of underlying vulnerabilities. A psychodynamic perspective (Blatt & Luyten, 2009) and a cognitive-behavioral perspective (Bieling et al., 2000) consistently distinguish between two types of vulnerability across development: one related to self-definition—encompassing concerns about self-worth, autonomy, achievement, feelings of inferiority and guilt, and achievement-related self-criticism—and another related to interpersonal aspects, including dependency, fear of rejection and abandonment, and concerns regarding relationships and social evaluation. These two dimensions manifest in different forms of internalizing psychopathology—especially in self-related and socially oriented forms of anxiety and depression—and this distinction is supported by empirical evidence (Marfoli et al., 2021).

Despite growing evidence supporting the multidimensionality of internalizing problems, longitudinal data spanning childhood to adolescence that demonstrate the reliability and development of these dimensions remain scarce. Among the available studies, Hankin et al. (2026) validated HiTOP-like dimensions in youth. Others have identified distinct internalizing dimensions in youth, including anxiety and fear, depression, and withdrawal components, further supporting a multidimensional structure (Cervin et al., 2020; Engler & Langer, 2025; Vedechkina et al., 2024). Cumulatively, this evidence informs our hypothesis that several dimensions underlie internalizing symptoms from childhood to adolescence; specifically, we expect to identify fear versus distress and self- versus socially oriented dimensions.

Regarding the trajectories of the level of these dimensions and potential sex-specific patterns, empirical evidence at the dimension level remains sparse, despite well-documented sex differences in aggregate internalizing trajectories (Kopala-Sibley et al., 2015). Existing evidence at the syndrome level offers a partial basis for hypotheses: depressive symptoms appear to increase comparably across boys and girls, whereas anxiety increases primarily in girls and may even decline in boys (Thorisdottir et al., 2017). At the level of psychological constructs, adolescence is a period in which the development of a coherent self-concept and a consolidated identity becomes the central developmental task (Alsaker & Kroger, 2020; Besser & Blatt, 2007; Harter, 2012), rendering concerns about self-definition and self-worth particularly salient; sex differences in this domain are modest but reliable; girls tend to report slightly lower general self-esteem (Gomez-Baya et al., 2016). In parallel, interpersonal contexts, such as peer relationships and emerging romantic partnerships, gain heightened significance, and markedly more so for girls (Pelletier-Baldelli et al., 2023; Rudolph, 2008): girls are more prone to interpersonal difficulties than boys, as they tend to generate more interpersonal stress, are more frequently exposed to it, and are more likely to translate that stress into psychopathology, particularly following pubertal onset (Rudolph & Conley, 2005). Together, these considerations lead us to expect levels on the identified dimensions to increase into adolescence, with sex-specific patterns emerging for each. In sum, we hypothesize that self-related and social concerns constitute distinct trajectories that both increase from childhood to adolescence, especially for girls, with a larger sex difference anticipated in the interpersonal dimension compared to the self-related dimension.

### Current Study

The present study had two primary aims: (a) to examine whether internalizing symptoms can be better explained by multiple latent dimensions capturing both fear vs. distress and self-oriented vs. socially oriented vulnerabilities, which may overlap or combine, through establishing MI across age and between sex; and (b) to track developmental changes in these dimensions from childhood to adolescence by sex, and to examine their continuity and cross-lagged effects across ages. For Aim (b), we hypothesized that levels on these dimensions would increase over time, and we expected a sex difference, especially during the transition into adolescence, with girls exhibiting elevated levels, particularly within the fear and social domains.

To address these aims, we used two longitudinal datasets that followed children across three important developmental stages: middle childhood, early adolescence, and mid-to-late adolescence. We identified longitudinal and sex-invariant dimensions, then estimated and compared their average-level trajectories across age for each sex. We then replicated the same procedure in a second dataset to assess whether the results would hold in a demographically different sample measured at comparable ages and with the same measures.

## Method

### Participants

This investigation utilized two independent longitudinal datasets, referred to as Sample A and Sample B throughout this manuscript. The samples differed notably in their sociodemographic characteristics, as detailed below.

#### Sample A

Participants were drawn from the Early Growth and Development Study (EGDS; Leve et al., 2019; 2026; Reiss et al., 2022), an ongoing longitudinal adoption study that tracks adopted children’s development along with characteristics of their birth and adoptive families (*N* = 561). Data were collected through home visits and online surveys beginning at 9 months of age. The adopted children (43% identified as female at birth) were placed in adoptive homes at a median age of 2.0 days (*M = 5.6* days, *SD = 11.3*). The majority of the children were identified as White (57%), with 11% identified as Black, 12% as Hispanic/Latinx, and the remaining 20% as multiracial or other ethnicities. Adoptive parents were, on average, 38.1 years old (*M = 38.1*, *SD = 5.7*) at the time of placement (Leve et al., 2019). Most adoptive parents (91%) identified as White and reported relatively high socioeconomic status, with a median household income of *$100-150*k when the children were 15/16 years old. Approximately 76% had completed at least four years of college or university education. The current analyses included participants with available data (*N = 488*), assessed at ages 7 (*N = 413*, *M = 7.04*, *SD = 0.18*), 11 (*N = 400*, *M = 11.21*, *SD = 0.49*), and 15/16 (*N = 294*, *M = 15.79*, *SD = 0.59*) years.

##### Sample A: Procedure

Recruitment for EGDS occurred through 33 adoption agencies across three U.S. regions—the Mid-Atlantic, the West/Southwest, and the Pacific Northwest—between 2003 and 2006. Eligibility criteria required that adoption placements were domestic, occurred within three months of birth, and involved adoptive parents who were not biologically related to the child. Children with major medical conditions (e.g., extreme prematurity) and families lacking English comprehension at an eighth-grade level were excluded (Leve et al., 2019). Data from age 15/16 were collected both before and during the COVID-19 pandemic. Of the available data, 53 participants completed in-home assessments prior to the pandemic (June 2019–March 2020), whereas 241 completed assessments remotely during the pandemic (129 between March 2020 and March 2021, and 112 after March 2021 when virtual visits were implemented).

##### Sample A: Missing Data

Missing data for children’s internalizing symptoms ranged from 16% to 40%, with the highest rate at ages 15/16. To assess potential bias from missingness, we conducted logistic regression analyses predicting missing data at each wave based on children’s sex, race, and prior internalizing symptoms. None of the predictors was statistically significant (*p*-values ranged from 0.32 to 0.99).

#### Sample B

Participants were drawn from the Child Representation and Regulation Project (ChiRRP), an ongoing, longitudinal study of children’s representation, regulation, and adaptation. Assessments involved parent-child dyads and began when the children were 4 years old (*N* = 250, 50% identified as female at birth; *M = 4.07* years, *SD = 0.23* years). The sample was ethnically and racially diverse (46% Latinx, 24.8% multiracial, 18% Black, and 11.2% White), and families represented a range of socioeconomic backgrounds (e.g., 30% were at or below the poverty line at age 7). Across waves, the participating caregivers were primarily biological mothers (91–94%), with other maternal figures (e.g., aunts, grandmothers, foster mothers) comprising 3–6.7% of the sample. About half of the caregivers were employed (51%), most were in a marital and/or cohabiting partnership (72%), and nearly half had completed secondary education or attended a technical school (46%). The sample was representative of the region where data were collected (U.S. Census Bureau, 2008). The current investigation used data collected at ages 7, 12, and 17 years, spanning middle childhood through late adolescence (*N = 222*). The age-specific assessments were conducted at 7 years (*N = 193*, *M = 7.13*, *SD = 0.23*), 12 years (*N = 194*, *M = 12.25*, *SD = 0.35*), and 17 years (*N = 183*, *M = 17.36*, *SD = 0.54*).

##### Sample B: Procedure

Recruitment was conducted by distributing flyers at community-based childcare centers. Families were excluded if the child had a diagnosed disability or developmental delay (*n=3*), or if the child lacked English comprehension (*n = 4*). Each assessment involved a three-hour laboratory visit. Caregivers were compensated with per assessment hour, and children received a small gift at ages 7 and 12 and *$40* per assessment hour at age 17. Data collection for age 17 began in October 2021.

##### Sample B: Missing Data

Missing data for internalizing symptoms ranged from 2% to 17% across waves, with the highest proportion occurring at age 17. To examine potential bias due to attrition, we conducted logistic regression analyses to test whether internalizing scores from the prior wave, sex, race, and poverty status (measured at age 7) predicted missingness at each wave. Results indicated that missingness was not significantly associated with the prior levels of internalizing symptoms or with any of the analyzed baseline predictors (*p*-values ranged from 0.14 to 0.99).

### Measures

#### Internalizing Symptoms

Both datasets included repeated administrations of the school-age version of the Child Behavior Checklist (CBCL/6–18; Achenbach & Rescorla, 2001) to assess children’s emotional and behavioral problems. Items were rated on a 3-point Likert scale (0 = *not true*, 1 = *somewhat or sometimes true*, 2 = *very true or often true*), with higher scores indicating greater symptom severity. Due to naturally low endorsement rates of symptoms in nonclinical samples, all items were treated as binary categories (i.e., presence or absence of the item). For this study, we focused on two subscales indexing internalizing symptoms: Anxious/Depressed (13 items) and Withdrawn/Depressed (8 items). One item (“talks about killing self”) was excluded from Sample B analysis due to a lack of variance at age 7, yielding a total of 20 items for this sample.

In Sample A, adoptive mothers completed the CBCL, with internal consistency ranging from *α =0.86* to *α =0.94*. In Sample B, caregivers (primarily maternal figures) rated children’s internalizing symptoms. These reports demonstrated acceptable internal consistency, with Cronbach’s alpha values ranging from *α =0.84* to *α =0.90*.

#### Sex

In both samples, sex assigned at birth was used as an independent variable. Data were limited to binary categories, which are referred to as “girls” and “boys” throughout this manuscript.

### Analytic Plan

Both samples were assessed at three time points spanning middle childhood (age 7), early adolescence (ages 11–12), and mid-to-late adolescence (ages 15/16–17)—-developmental periods during which sex differences in internalizing symptoms typically emerge and intensify (Costello et al., 2011; Keyes & Platt, 2024; Zahn-Waxler et al., 2008). The slight age differences between samples reflect the predetermined schedules of the parent studies.

All analyses were conducted separately for each sample, with Sample A serving as the primary analysis and Sample B as an independent replication. Most analyses were performed in R (Version 4.4.1; R Core Team, 2024) using the lavaan package (Version 0.6-18; Rosseel, 2012). ESEM models, not supported in lavaan, were estimated in Mplus 8.7 (Muthén & Muthén, 1998-2017) with Geomin oblique rotation (Asparouhov & Muthén, 2009). Both ESEM and confirmatory factor analysis (CFA) models used the WLSMV estimator (Flora & Curran, 2004), appropriate for ordinal data, employing a polychoric correlation matrix with threshold parameterization. Nested model comparisons used the DIFFTEST procedure in Mplus and lavTestLRT with the satorra.2000 correction in lavaan (Satorra & Bentler, 2010). Given that missingness was not significantly predicted by observed variables (see Participants), analyses used pairwise available data, which is appropriate under missingness completely at random (MCAR). The analytic strategy proceeded in three steps.

#### Step 1. Longitudinal Factor Structure

We examined the factor structure of internalizing symptoms across age using longitudinal ESEM (Asparouhov & Muthén, 2009), which allows all items to load on all factors without constraining cross-loadings to zero, making the solution more data-driven than standard CFA. We tested one- through four-factor models, keeping the number of factors identical across age to maintain a consistent configural structure. Each age was treated as a separate exploratory factor analysis (EFA) block, with within-time and cross-age factor correlations freely estimated, and item residuals correlated across all age pairs to account for repeated measures. Factor variances and intercepts were fixed to 1 and 0, respectively. Model fit was evaluated using RMSEA *≤ * 0.06 and CFI/TLI *≥ * 0.95 as good fit, and RMSEA *≤ * 0.08 and CFI/TLI *≥ * 0.90 as acceptable (Cheung & Rensvold, 2002; Zheng & Bentler, 2025), alongside the interpretability and reliability of the extracted factors.

#### Step 2. Measurement Invariance

Using the optimal factor solution from Step 1, we tested longitudinal MI via ESEM following the sequence recommended by Widaman et al. (2010): weak MI (equal loadings across age) followed by strong MI (additionally constraining thresholds). Nested model comparisons were evaluated using *Δ *CFI < 0.01 as the criterion for acceptable fit deterioration (Cheung & Rensvold, 2002; Putnick & Bornstein, 2016); scaled $\chi ^2$ difference tests were also reported but, given their sensitivity to sample size, treated as supplementary. Between-sex MI was subsequently tested using CFA (ESEM between-sex models did not converge), retaining only conceptually meaningful and large loadings *≥ * 0.35; below this threshold, loadings are generally considered non-meaningful (Costello & Osborne, 2005).

#### Step 3. Factor Trajectories and Cross-Lagged Paths

Factor mean intercepts from the optimal between-sex MI model were used to examine mean-level developmental change in each factor. Pairwise changes across adjacent ages and between-sex differences at each age were tested using Wald tests (lavTestWald). Finally, to examine prospective associations between factors while controlling for prior levels, autoregressive and cross-lagged models were estimated by replacing cross-age correlations with regression paths across adjacent ages.

**Fig. 1:**
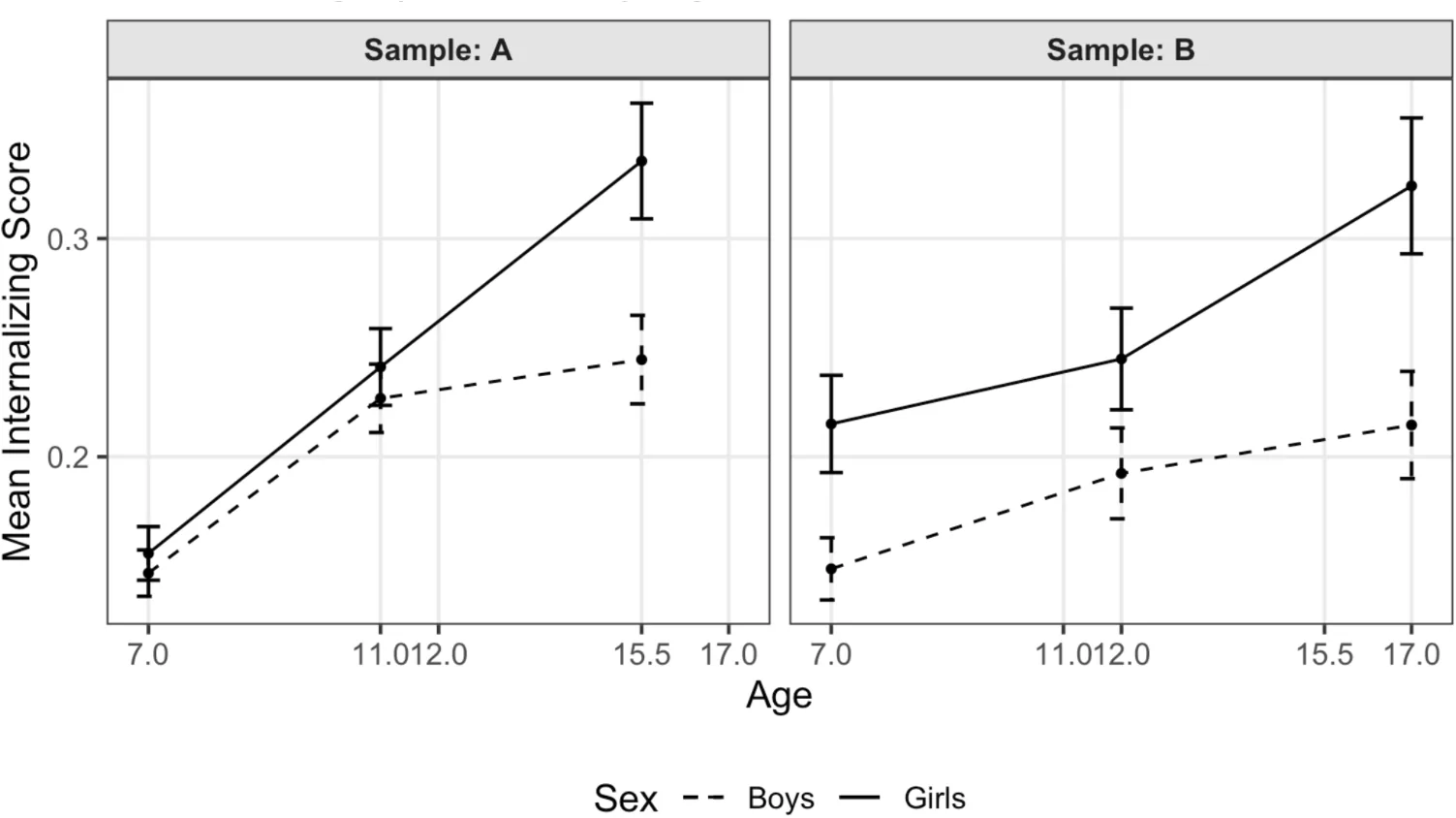
Mean internalizing symptom scores by age, sex, and sample. *Note.* Internalizing symptoms reflect the average of the Anxious/Depressed and Withdrawn/Depressed CBCL subscales. Error bars represent ±1 standard error of the mean

## Results

### Symptoms Average Trends

The average level trends of internalizing symptom scores across age, sex, and sample are presented in Fig. 1. Overall, mean internalizing scores increased with age in both samples. Given that scores theoretically range from 0 to 2, levels were generally low. In Sample A, mean scores increased from approximately 0.15 at age 7 for both sexes to 0.34 for girls and 0.24 for boys at age 15/16. In Sample B, a similar pattern was observed, with average internalizing symptoms increasing from 0.22 at age 7 to 0.32 at age 17 for girls and from 0.15 at age 7 to 0.21 at age 17 for boys.

Sex differences at each age were tested using Welch *t*-tests (assuming unequal variances). In both samples, girls were rated as having higher internalizing symptoms by late adolescence. However, in Sample A, sex differences were negligible at ages 7 (*t(375.1) = 0.56*, *p =0.57*) and 11 (*t(370.4) = 0.61*, *p =0.54*) but widened progressively with age (at age 15/16: *t(256.2) = 2.73*, *p=0.007*). In Sample B, a sex gap was already present at ages 7 (*t(158) = 2.51*, *p =0.01*), marginally present at age 12 (*t(165.2) = 1.94*, *p =0.054*) and widened further by age 17 (*t(148.3) = 2.70*, *p = 0.008*).

At age 15/16 in Sample A, data collection spanned the COVID-19 pandemic across three assessment conditions: in-person pre-pandemic (*n = 53*, *M = 0.36*), remote at pandemic onset (*n=129*, *M = 0.25*), and virtual later into the pandemic (*n=112*, *M = 0.29*). A one-way ANOVA indicated a marginal effect of assessment status, *F(2, 291) = 2.68*, *p =0.070*, with internalizing symptoms appearing lower in remote assessments compared to in-person, suggesting no clear evidence of an escalation in symptoms due to COVID-19, though these comparisons are limited by the small pre-pandemic group size (*n = 53*) and unequal group sizes.

### Factor Structure and Measurement Invariance

#### Sample A

Longitudinal ESEMs across ages 7, 11, and 15/16 were estimated with one- through four-factor solutions. Model fit improved substantially from one to three factors, but the improvement from three to four factors fell below the recommended threshold (i.e., *Δ *CFI *<0.01*). We therefore selected the three-factor model as the configural basis (see Table 1 for fit indices and model comparisons). Next, within the ESEM framework, we tested the weak model. Weak MI (equal loadings across age) was supported, $\Delta \chi ^2(108) = 120.81$, *p =0.19*. Strong MI (additionally equal thresholds) showed acceptable practical change in fit (*Δ *CFI *= -0.005*, *Δ *RMSEA) with excellent global fit ($\chi ^2(1827) = 1934.85$, *p =0.04*, CFI *=0.982*, RMSEA *=0.011*), and was retained. The three factors were named low self-worth/depression (LSW/Dep), social/general anxiety (S/GA), and depression/withdrawal (Dep/With) (see Table 2 for details).

Retaining longitudinal strong MI across age and the three-factor structure from the previous step, we next tested between-sex MI. Because the ESEM model did not converge, we switched to a CFA model (see Analytic Plan). We began with the configural model, in which loadings and thresholds were estimated separately for girls and boys (while constrained equal across age). We then tested weak MI by constraining loadings, followed by strong MI by additionally constraining thresholds. All models showed good fit, and constraints did not result in a meaningful decline in fit (Table 3). Thus, the strong MI model was retained as the optimal model based on parsimony, $\chi ^2_\text {scaled}(3760) = 3959.19$, *p =0.01*, CFI *=0.961*, TLI *=0.960*, RMSEA *=0.015*, $\Delta \chi ^2_\text {scaled}(18) = 27.68$, *p =0.13*, *Δ *CFI *= -0.001*, and *Δ *RMSEA *<0.001*.

**Table 1:** Longitudinal ESEM model fit indices for Sample A and Sample B

Sample	Model	*df*	$\chi ^2_\text {scaled}$	*p*	CFI	TLI	RMSEA	$\Delta \chi ^2$	*Δ df*	*Δ *CFI
A	1-factor	1824	2207.00	<0.001	0.935	0.931	0.021	—	—	—
	2-factor	1755	1970.41	<0.001	0 .968	0.964	0.015	236.59^*^	69	0.033
	3-factor$^{\dagger }$	1683	1764.00	0.08	0.986	0.984	0.007	178.79^*^	72	0.018
	4-factor	1608	1637.72	0.31	0.994	0.991	0.004	126.28^*^	75	0.008
A	Configural	1683	1764.00	0.08	0.986	0.984	0.007	—	—	—
	Weak	1791	1869.00	0.10	0.987	0.986	0.009	120.81	108	<0.001
	Strong$^{\dagger }$	1827	1934.85	0.04	0.982	0.981	0.011	133.45^*^	36	-0.005
B	1-factor	1647	1775.85	0.01	0.940	0.936	0.019	—	—	—
	2-factor	1581	1669.11	0.06	0.959	0.954	0.016	107.23^*^	66	0.019
	3-factor$^{\dagger }$	1512	1571.23	0.14	0.973	0.968	0.013	107.68^*^	69	0.014
	4-factor	1440	1472.34	0.27	0.985	0.982	0.010	103.99^*^	72	0.012
B	Configural	1512	1571.23	0.14	0.973	0.968	0.013	—	—	—
	Weak	1614	1672.22	0.15	0.973	0.970	0.013	117.25	102	<0.001
	Strong	1648	1737.87	0.06	0.958	0.955	0.016	154.01^*^	34	–0.015
	Partial strong$^{\dagger }$	1646	1721.48	0.10	0.965	0.962	0.014	106.05^*^	32	–0.008

*Note.* All models were estimated with WLSMV

$\chi ^2_s$ scaled chi-square, *CFI* comparative fit index, *TLI* Tucker–Lewis index, *RMSEA* root mean square error of approximation

*Δ * indices reflect change relative to the previous model in each sequence (except the partial strong model, which is compared to the weak model)

$^{\dagger }$ indicates the selected model in the analysis$. ^{*}p < 0.05$

**Table 2:** ESEM factor loadings and item thresholds from the longitudinal (Partial) strong measurement invariance models

		Sample A				Sample B			
Item	Description	Low Self-Worth / Depression	Social/General Anx.	Depression/ Withdrawal	Threshold	Low Self-Worth/ Depression	Social Anx./ Withdrawal	Factor 3	Threshold
5	Very little to enjoy	0.47	–	0.46	1.48	0.39	–	**0.54**	1.36
14	Cries a lot	0.39	–	–	1.26	0.46	–	–	0.45$^{*}$
29	Fears animals/situations	–	**0.74**	–	0.78	–	0.42	–	0.93
30	Fears school	–	**0.63**	–	1.66	**0.67**	–	–	1.64
31	Fears doing bad things	**0.52**	0.42	–	1.19	0.37	0.42	–	1.49
32	Feels must be perfect	0.41	–	–	0.52	–	**0.64**	–	0.51
33	Feels unloved	**0.82**	–	–	1.14	**0.95**	–	-0.36	1.06
35	Feels worthless/inferior	**0.72**	–	–	1.05	**0.90**	–	–	1.39
42	Likes to be alone	–	–	**0.51**	0.99	–	**0.53**	–	0.93
45	Nervous/high strung/tense	–	**0.62**	–	0.92	–	–	–	0.91
50	Too fearful or anxious	–	**0.76**	–	0.90	–	0.36	–	1.24
52	Feels too guilty	–	**0.54**	–	1.58	–	**0.55**	–	1.64
65	Refuses to talk	–	–	0.39	1.38	–	0.35	–	1.25
69	Secretive/keeps things to self	–	–	0.36	1.05	–	0.36	–	0.73
71	Self-conscious/embarrassed	–	0.45	0.35	0.51	–	**0.78**	–	0.14
75	Shy/timid	–	**0.59**	–	1.02	–	**0.86**	–	0.58
91	Talks about killing self	**0.90**	–	–	1.88	–	–	–	–
102	Underactive/slow/no energy	–	–	**0.46**	1.47	0.41	–	–	1.22
103	Unhappy/sad/depressed	**0.77**	–	–	1.33	**0.68**	–	–	1.09
111	Withdrawn/not involved	–	–	**0.68**	1.84	–	**0.53**	0.42	1.65
112	Worries	–	**0.56**	–	0.45	–	**0.63**	–	0.58

*Note.* Factor loadings and thresholds are constrained equal across ages under strong measurement invariance. For Sample A, ages are 7, 11, and 15/16. For Sample B, ages are 7, 12, and 17. Loadings with *|λ | < 0.35* are suppressed (–); loadings with $\lambda \ge 0.50$ are bolded. Factor 3 in Sample B was not retained due to insufficient reliability. Item 91 (Talks about killing self) was excluded from Sample B analyses due to zero endorsement at age 7. $^{*}$Item 14 threshold was freely estimated across ages in Sample B (partial strong MI); threshold at age 7 *=0.45*, age 12 *= 1.26*, and age 17 *= 1.69*

#### Sample B

Parallel analyses were conducted across ages 7, 12, and 17 for one- through four-factor solutions. Although fit continued to improve through four factors (see Table 1), we retained the three-factor model as the configural basis, as the fourth factor became unstable and unreliable, with no loadings above 0.25. Weak MI was supported with negligible change in fit (*Δ *CFI *<0.001*). The scaled $\chi ^2$ difference test was nonsignificant ($\Delta \chi ^2(102) = 117.25$, *p = 0.14*), and given the negligible *Δ *CFI, weak MI was retained. Strong MI showed a decline in fit beyond the acceptable level (*Δ *CFI *= -0.015*). Inspection of modification indices indicated that freeing the threshold of one item (“crying a lot”) across ages would yield the greatest improvement; doing so resulted in a partial strong MI model with acceptable fit (relative to the weak model: *Δ *CFI *= -0.008*, *Δ *RMSEA *= 0.001*; $\chi ^2_\text {scaled}(1646) = 1721.48$, *p =0.10*, CFI *=0.965*, RMSEA *=0.014*). Two factors were well-defined and labeled LSW/Dep and Social Anxiety/Withdrawal (SA/With) (see Table 2). A third factor emerged but was poorly identified, with only two primary loadings and substantial cross-loadings, and was therefore not labeled or retained for subsequent analyses.      

Next, we tested between-sex MI. As in Sample A, the ESEM model did not converge, so we switched to a CFA model (see Analytic Plan), retaining the two well-defined factors. Sequential tests of configural, weak, and strong MI indicated acceptable fit for all models (Table 3). The strong MI model was retained as the optimal model, $\chi ^2_\text {scaled}(3109) = 3243.80$, *p =0.04*, CFI *=0.931*, TLI *=0.929*, RMSEA *=0.020*, $\Delta \chi ^2_\text {scaled}(19) = 30.16$, *p =0.05*, with minimal change relative to the weak model (*Δ *CFI *= -0.001*, *Δ *RMSEA *<0.001*).

**Table 3:** Measurement invariance across sex: Samples A and B

			Fit Indices				*Δ * from Previous			
Sample	Model	*df*	$\chi ^2_\text {scaled}$	CFI$_s$	TLI$_s$	RMSEA$_s$	SRMR	$\Delta \chi ^2$	*Δ df*	*Δ *CFI
Sample A (girls *n = 211*, boys *n = 277*)										
	1. Configural	3722	3927.30$^{*}$	0.960	0.958	0.015	0.165	—	—	—
	2. Weak	3742	3939.12 $^{*}$	0.962	0.960	0.015	0.168	$19.78^{n.s.}$	20	
	3. Strong$^{\dagger }$	3760	3959.19$^{*}$	0.961	0.960	0.015	0.168	$27.68^{n.s.}$	18	–0.001
Sample B (girls *n = 107*, boys *n = 115*)										
	1. Configural	3072	3210.29$^{*}$	0.929	0.927	0.020	0.215	—	—	—
	2. Weak	3090	3222.86$^{*}$	0.932	0.930	0.020	0.217	$19.34^{n.s.}$	18	0.003
	3. Strong$^{\dagger }$	3109	3243.80$^{*}$	0.931	0.929	0.020	0.217	$30.16^{*}$	19	–0.001

*Note.* All models estimated with WLSMV

$\Delta \chi ^2$ = scaled chi-square difference test. *CFI* comparative fit index, *TLI* Tucker–Lewis index, *RMSEA* root mean square error of approximation, *SRMR* standardized root mean square residual. *Δ *CFI *≤ 0.010* and *Δ *RMSEA *≤ 0.015* indicate acceptable invariance

$$^{\dagger }$$ optimal model

$$^{*}p < 0.05$$ $$^{n.s.}p > 0.05$$ for scaled $$\chi ^2$$

### Factor Trajectories and Sex Effects

**Fig. 2:**
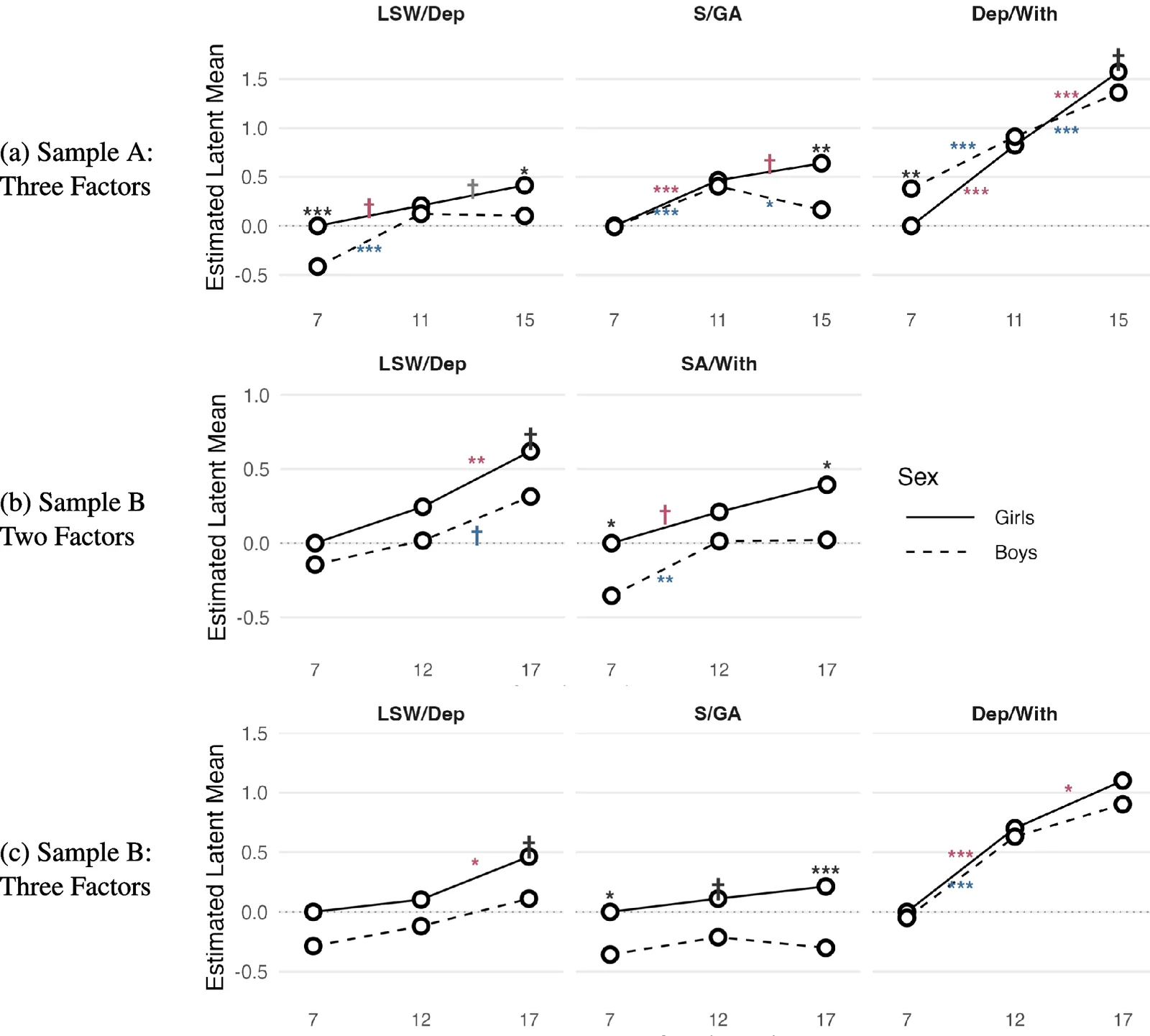
Latent factor mean trajectories across development for girls and boys in Sample A and Sample B. *Note.* Factor means are expressed as deviations from girls’ age 7 mean, fixed to zero as the identification reference. Models estimated under (partial) strong MI across age and sex. Symbols above age points indicate sex differences at that age; symbols above connecting segments indicate temporal change within sex. *†* *p <0.10*, * *p <0.05*, ** *p <0.01*, *** *p <0.001*. Note that *y*-axis scales differ across panels

Figure 2 depicts the age trends in estimated factor means for each sex across Sample A (panel a) and Sample B (panel b). For comparison, the trajectories of a three-factor solution for Sample B with the same structure as Sample A were also estimated and depicted (panel c). This comparison model also yielded acceptable fit, $\chi ^2_\text {scaled}(3050) = 3179.53$, *p =0.05*, CFI *=0.923*, TLI *=0.920*, RMSEA *=0.021*, 90% CI [0.000, 0.030].

#### Sample A

Among girls, all three factors increased significantly or marginally from age 7 to age 11 (LSW/Dep: $\chi ^2(1) = 3.78$, *p =0.052*; S/GA: $\chi ^2(1) = 23.41$, *p <0.001*; Dep/With: $\chi ^2(1) = 28.17$, *p <0.001*). From age 11 to age 15/16, LSW/Dep ($\chi ^2(1) = 3.05$, *p =0.08*) and S/GA ($\chi ^2(1) = 3.69$, *p =0.055*) showed marginal increases, whereas Dep/With continued to increase ($\chi ^2(1) = 27.48$, *p <0.001*).

Among boys, all three factors increased significantly from age 7 to age 11 (LSW/Dep: $\chi ^2(1) = 32.01$, *p <0.001*; S/GA: $\chi ^2(1) = 17.68$, *p <0.001*; Dep/With: $\chi ^2(1) = 16.81$, *p <0.001*). However, from age 11 to age 15/16, LSW/Dep showed almost no change ($\chi ^2(1) = 0.19$, *p =0.66*), S/GA declined significantly ($\chi ^2(1) = 4.86$, *p =0.03*), and Dep/With continued to increase ($\chi ^2(1) = 15.78$, *p <0.001*).

Regarding sex differences, at age 7 girls showed higher LSW/Dep ($\chi ^2(1) = 10.79$, *p =0.001*), no sex difference in S/GA ($\chi ^2(1) = 0.04$, *p =0.83*), and lower Dep/With ($\chi ^2(1) = 7.13$, *p =0.008*) than boys. No significant sex differences were found at age 11 (all *p*s *>0.37*). By age 15/16, girls showed significantly higher LSW/Dep ($\chi ^2(1) = 5.56$, *p =0.02*) and S/GA ($\chi ^2(1) = 9.03$, *p =0.003*) than boys, with no significant difference in Dep/With ($\chi ^2(1) = 2.80$, *p =0.09*).

#### Sample B

Girls showed no significant change from age 7 to age 12 in LSW/Dep ($\chi ^2(1) = 2.56$, *p =0.11*), and marginal change in SA/With ($\chi ^2(1) = 3.18$, *p =0.08*). From age 12 to age 17, however, LSW/Dep increased significantly ($\chi ^2(1) = 9.18$, *p =0.002*), whereas SA/With did not change ($\chi ^2(1) = 2.57$, *p =0.11*).

Boys showed a distinct pattern. From age 7 to age 12, LSW/Dep did not change ($\chi ^2(1) = 1.38$, *p =0.24*) whereas SA/With increased significantly ($\chi ^2(1) = 10.28$, *p =0.001*). From age 12 to age 17, LSW/Dep increased marginally ($\chi ^2(1) = 3.65$, *p =0.06*), whereas SA/With showed no significant change ($\chi ^2(1) = 0.01$, *p =0.94*).

Between sexes, patterns differed by age. At age 7, girls and boys did not differ significantly on LSW/Dep ($\chi ^2(1) = 0.68$, *p =0.41*), but girls had higher mean levels of SA/With ($\chi ^2(1) = 5.02$, *p =0.03*). At age 12, no sex differences emerged (LSW/Dep: $\chi ^2(1) = 1.58$, *p =0.21*; SA/With: $\chi ^2(1) = 1.38$, *p =0.24*). By age 17, girls were marginally higher than boys on LSW/Dep ($\chi ^2(1) = 3.00$, *p =0.08*) and significantly higher on SA/With ($\chi ^2(1) = 5.11$, *p =0.02*).

#### Sample B: Three-Factor (Comparison Solution)

Girls’ LSW/Dep did not change from age 7 to age 12 ($\chi ^2(1) = 0.39$, *p =0.53*), but increased significantly from age 12 to age 17 ($\chi ^2(1) = 5.95$, *p =0.01*). S/GA did not change significantly across either interval (all *p*s *>0.37*). Dep/With increased significantly from age 7 to age 12 ($\chi ^2(1) = 16.87$, *p <0.001*) and continued to increase, though with a somewhat slower slope, through age 17 ($\chi ^2(1) = 5.81$, *p =0.02*).

Boys’ LSW/Dep did not change from age 7 to age 12 ($\chi ^2(1) = 1.42$*, p =0.23*), or from age 12 to age 17 ($\chi ^2(1) = 2.08$, *p =0.15*). S/GA fluctuated, but non-significantly, across intervals (all *p*s *>0.30*). Dep/With increased significantly from age 7 to age 12 ($\chi ^2(1) = 19.15$, *p <0.001*), but not further through age 17 ($\chi ^2(1) = 2.69$, *p =0.10*).

Regarding sex differences, girls showed marginally higher LSW/Dep by age 7 ($\chi ^2(1) = 2.77$, *p =0.10*) and non-significantly higher levels at age 12 (*p =0.24*), and were marginally higher by age 17 ($\chi ^2(1) = 3.65$, *p =0.06*). Girls also showed higher S/GA than boys at ages 7 and 17

 (age 7: $\chi ^2(1) = 4.51$, *p =0.03*; age 12: $\chi ^2(1) = 3.39$, *p =0.07*; age 17: $\chi ^2(1) = 8.35$, *p =0.004*). Dep/With did not differ between sexes at any age (all *p*s *>0.33*).

### Factor Correlations and Cross-Lagged Effects

Within each age, all factor pairs were significantly and positively correlated across both samples and both sexes (all *p*s *<0.001*), with correlations ranging from moderate to strong (*r =0.46-0.85*) in Sample A and strong (*r =0.63-0.87*) in Sample B, and generally increasing with age. One exception was boys at age 7, where the LSW/Dep–SA/With correlation exceeded unity (*r = 1.06*), a Heywood case likely reflecting unstable polychoric correlation estimates due to low symptom endorsement rates at that age.

#### Sample A

Autoregressive paths indicated consistent stability across intervals for LSW/Dep in girls (*b = 0.77-0.79*, all *p*s *<0.001*) and boys (*b = 0.69-0.97*, all *p*s *≤ 0.018*). S/GA was stable across both intervals in girls (*b = 0.82-0.86*-, all *p*s *<0.001*), but only in the later interval (from age 11 to 15/16) in boys (*b = 0.51*, *p =0.04*), with no significant stability from age 7 to 11 (*b = 0.30*, *p =0.27*). Dep/With was stable from age 7 to 11 in both sexes (girls: *b = 0.80*, *p =0.02*; boys: *b = 0.67*, *p =0.03*), but only in boys from age 11 to 15/16 (*b = 0.65*, *p =0.004*), with a non-significant path for girls (*b = 0.26*, *p =0.11*).

Cross-lagged paths were largely non-significant, with some exceptions. LSW/Dep at age 7 predicted S/GA at age 11 in boys (*b = 0.70*, *p =0.001*) and marginally in girls (*b = 0.34*, *p =0.08*); Dep/With at age 11 marginally predicted lower S/GA at age 15/16 in girls (*b = -0.26*, *p =0.06*).

#### Sample B

Age 7 to 12 autoregressive paths showed large standard errors and were non-significant for both factors and both sexes (all *p*s *>0.16*), likely reflecting estimation instability given the small sample. From age 12 to 17, LSW/Dep showed stability in girls (*b = 0.47*, *p =0.003*), but not in boys (*b = 0.21*, *p =0.44*). SA/With showed significant stability in both girls (*b = 0.54*, *p <0.001*) and boys (*b = 0.55*, *p =0.048*). Cross-lagged paths were non-significant, with one marginal exception: SA/With at age 12 marginally predicted LSW/Dep at age 17 in boys (*b = 0.35*, *p =0.08*). 

## Discussion

In this study, we aimed to identify internalizing dimensions in childhood that remain stable across age and between sexes, to obtain reliable developmental trajectories and make meaningful group comparisons. We used longitudinal data from two demographically different samples — adopted children from higher-SES families and biological children from lower-SES families — covering middle childhood (age 7), early adolescence (age 11 or 12), and adolescence (age 15/16 or 17). Consistent with previous research (Keyes & Platt, 2024), both samples showed similar age and sex patterns: internalizing symptoms increased from childhood to adolescence, more strongly for girls starting in early adolescence. This increase is not attributable to COVID-19 — in Sample A, pandemic assessments showed slightly lower symptom levels than pre-pandemic, and in Sample B the age-17 assessment began in October 2021, after the acute phase.

### Factor Structure of Internalizing Symptoms

Although average-level trends are informative, they do not reveal the distinct underlying vulnerabilities that may follow different developmental pathways. Consistent with previous research (Engler & Langer, 2025; Kotov et al., 2017), our results supported a multidimensional structure. We used ESEM to identify data-driven dimensions that retain a similar meaning across age, without imposing a specific theoretical model of symptom organization. In Sample A, we found dimensions of LSW/Dep, S/GA, and Dep/With. However, in Sample B, S/GA and Dep/With combined into a single SA/With factor. These results are broadly consistent with a growing literature reporting withdrawal as a dimension of internalizing symptoms beyond anxiety and depression (Vedechkina et al., 2024).

The low self-worth/depression factor showed the highest loadings on items such as feeling unloved, worthless, inferior, unhappy, sad, anhedonia, and crying. Strong longitudinal MI was supported for these items from age 7 through adolescence, and across both sexes in both samples. There was one minor exception: the threshold for crying changed across ages in Sample B, in a theoretically justifiable direction, with older children crying less frequently at comparable levels of distress.

This factor broadly aligns with the distress subfactor in the HiTOP framework (Krueger & Eaton, 2015; Watson et al., 2022), but places greater emphasis on self-worth problems as core features of distress during this developmental period. In the literature, low self-worth is often conceptualized either as a cause of depression (e.g., the vulnerability model; Orth & Robins, 2013) or as a consequence of depression (e.g., the scar model; Manna et al., 2016). Although our study was not designed to adjudicate between these possibilities, we did not find clear evidence that one precedes the other within this developmental window. Instead, parent-reported self-worth problems and depressed mood appeared to co-occur strongly, suggesting that they may reflect two manifestations of a common underlying vulnerability from as early as age 7. This dimension may correspond to a subtype of depression characterized as introjective or self-critical depression (Blatt & Luyten, 2009), or the autonomy-related subtype (Bieling et al., 2000), depending on the clinical framework. The strong link between self-worth and depressed mood is consistent with a large meta-analysis of over 270,000 adolescents (Yeo et al., 2023), which found that self-esteem had a strong average correlation with depression (*r =0.52*) and that this relationship is bidirectional.

Item thresholds within this factor were not uniform: feeling unloved and worthless had lower thresholds, whereas anhedonia and suicidal ideation had the highest, suggesting that self-worth problems may be more observable to caregivers at lower levels of depression. If self-worth problems represent an early and stable marker of depressive vulnerability, this underscores the importance of early identification rather than waiting for more severe symptoms to emerge.

The second dimension reflected anxiety and fear symptoms, especially social anxiety — with the highest loadings on shyness, self-consciousness, embarrassment, worrying, fearfulness, and nervousness — showing strong longitudinal MI from age 7 through adolescence for both sexes in both samples. This dimension aligns with the fear subfactor in HiTOP (Krueger & Eaton, 2015; Watson et al., 2007), but with a stronger emphasis on social-evaluative content than the broader phobic-fear material typically forming that subfactor. This social focus is consistent with developmental temperament research on the longitudinal continuity between early childhood behavioral inhibition, i.e., fearfulness toward novel and social situations, and later social anxiety, and suggests that by middle childhood the fear dimension is already loaded toward social-evaluative content (Chronis-Tuscano et al., 2009; Hirshfeld-Becker et al., 2008; Nelemans et al., 2019).

Item thresholds also differed within this factor: self-consciousness, embarrassment, and worry had relatively lower thresholds, whereas withdrawal behaviors and guilt had higher thresholds, suggesting these latter symptoms appear only at higher severity or may be harder for parents to notice.

The two samples differed in whether social anxiety and social withdrawal formed a single dimension or two separate ones. Social withdrawal can be an outcome of social anxiety, but it can also be benign or even protective as a coping strategy in early childhood (Asendorpf, 1990; Coplan et al., 2018). However, it may become more associated with depression as social demands increase during adolescence (Coplan et al., 2018) — particularly through peer exclusion and loneliness — potentially consolidating into a distinct depressive dimension (Bieling et al., 2000; Blatt & Luyten, 2009). In our data, the increase in withdrawal was steeper in Sample A than Sample B, which may partly explain why a distinct Dep/With factor emerged in that sample. Beyond developmental dynamics, cultural background may further weaken the withdrawal–depression link. Sample A’s adoptive parents were 91% White, whereas Sample B’s biological families were considerably more racially and ethnically diverse (46% Latinx, 18% Black), and withdrawal may be a less viable coping option in family cultures emphasizing interdependence. In addition, the two samples differed substantially in family context; chronic family-level stress (e.g., poverty) in Sample B may make withdrawal function less as a distinct depressive process and more as a context-driven response that overlaps with social-evaluative concerns. More research is needed to clarify when and for whom social withdrawal differentiates into a distinct depressive dimension.

In addition to the dimension-level differences between the two samples, item-level loadings also differed: in Sample B, perfectionism and self-consciousness anchored the SA/With factor most strongly, fears of school shifted toward LSW/Dep, and withdrawal behaviors cross-loaded rather than forming a clean anhedonia/withdrawal anchor as in Sample A. Thus, although Sample B recovers the broad LSW/Dep and SA/With dimensions identified in Sample A, different items most strongly define each, making it broadly consistent with, rather than a strict replication of, the Sample A structure.

The finding of strong MI across sex in both samples is important. Some earlier studies have reported sex-related item bias, mostly in self-report and clinical samples, and often under stricter criteria (Fonseca-Pedrero et al., 2010; Keller et al., 2020; van der Sluis et al., 2017; Wu & Huang, 2014). Our use of caregiver reports in community samples may explain this difference, consistent with other studies that found full scalar invariance across sex in similar contexts (Brunet et al., 2014; Jovanović, 2025; Shabani et al., 2025; Silva et al., 2024). Caregiver reports may reduce the detection of sex-specific bias, especially at lower levels of symptoms, as parents may be less sensitive to subtle sex-related differences in how adolescents express distress.

Compared to the original CBCL structure — which defines two internalizing subscales (in addition to somatic complaints), Anxious/Depressed and Withdrawn/Depressed (Achenbach & Rescorla, 2001) — our results revealed meaningful structural differences. A three-factor solution, splitting Anxious/Depressed into LSW/Dep and S/GA, provided better fit, consistent with Vedechkina et al. (2024). Moreover, even in the two-factor solution, social anxiety items clustered with withdrawal rather than with LSW/Dep, suggesting that the Anxious/Depressed subscale conflates two distinct dimensions. In our analysis, depressive symptoms did not emerge as a single unified dimension, consistent with the CBCL’s own split structure — pointing to at least two developmental pathways: one linked to self-worth from early childhood, and another tied to social concerns in adolescence. Notably, the three Sample A factors — LSW/Dep, a cognitive/self-evaluative depressed-mood factor; S/GA, a social and generalized anxiety factor; and Dep/With, a low-energy, anhedonic, withdrawal factor — map more closely onto DSM-5-TR criterion-level groupings than does the original CBCL Anxious/Depressed vs. Withdrawn/Depressed split. Specifically, LSW/Dep and Dep/With correspond to the affective-cognitive (depressed mood, worthlessness) and anhedonic-somatic (loss of interest, fatigue) criterion clusters of major depressive disorder, respectively (Tsai et al., 2014), while S/GA aligns with the social anxiety and generalized anxiety disorder symptom clusters (American Psychiatric Association, 2022).

Item groupings also differed from the original CBCL. Notably, unhappiness and sadness loaded onto LSW/Dep in our results, whereas the CBCL places them under Withdrawn/Depressed. This likely reflects a methodological constraint in the CBCL: because anhedonia is assigned only to the Withdrawn/Depressed subscale, unhappiness and sadness — which co-occur with anhedonia — are pulled into that subscale as well. In our ESEM, however, anhedonia was free to cross-load on both LSW/Dep and withdrawal, and did so meaningfully on both. Once this constraint was relaxed, unhappiness and sadness loaded more strongly onto LSW/Dep, possibly suggesting they are more closely tied to self-evaluative distress than to social withdrawal.

### Factor Trajectories and Cross-Lagged Effects

Consistent with our hypotheses and prior work (Keyes & Platt, 2024; Zahn-Waxler et al., 2008), dimensions generally increased from middle childhood to early adolescence in both sexes and samples, but trajectories diverged during adolescence. For LSW/Dep, both sexes continued to increase, but more strongly in girls, leading to a sex difference by late adolescence, significant in Sample A and marginal in Sample B — consistent with evidence that girls show more identity-related concerns and self-critical thinking after puberty (Gentile et al., 2009; Harter, 2012; Kopala-Sibley et al., 2015).

For S/GA, the sex difference was more pronounced: girls continued to increase from early to late adolescence, whereas boys showed a decrease in Sample A and almost no change in Sample B. The increase in girls is consistent with findings that they are more sensitive to social evaluation and more likely to translate interpersonal stress into anxiety (Rudolph & Conley, 2005). The decrease in boys may reflect gender norms discouraging anxiety expression, social adjustment over time, reduced parental visibility as adolescents seek independence (Zahn-Waxler et al., 2008), or a genuine developmental reduction in social-evaluative fears. Dep/With increased for both sexes in both samples, with no sex differences in Sample B and only a difference at age 7 in Sample A, where boys were higher. Of the three factors, S/GA most clearly drove the emerging sex gap in internalizing symptoms during adolescence.

Autoregressive paths in both samples indicated generally strong homotypic continuity within each dimension, suggesting rank-order stability across development (Gazelle & Rubin, 2019; Magson et al., 2022), with one exception: for girls, Dep/With in adolescence was not significantly predicted by early adolescent levels, possibly because the sharp concurrent increase in this dimension outweighed earlier individual differences. Cross-lagged paths were mostly non-significant, suggesting limited directional influence between dimensions. Two exceptions emerged in Sample A: LSW/Dep at age 7 predicted S/GA at age 11 particularly for boys, and Dep/With weakly predicted S/GA for girls in late adolescence. In Sample B, effects were weaker and non-significant, likely reflecting the smaller sample. Overall, internalizing dimensions showed strong homotypic but limited heterotypic continuity, with cross-dimensional effects depending on developmental stage and sex.

### Strengths, Limitations, and Future Directions

Our study had several strengths. We utilized two independent longitudinal datasets that followed children from childhood to adolescence. These samples differed in important ways: Sample A comprised adopted children from more affluent families, whereas Sample B included biological families from low-income backgrounds. Cross-validation across these distinct groups strengthens the reliability, reproducibility, and generalizability of our findings. Furthermore, the study leveraged a relatively wide age range, allowing us to track developmental patterns across the key transition from childhood to adolescence.

Our study also had several limitations. First, as in many long-term longitudinal studies, we faced attrition, which reduced statistical power at later ages. Second, we relied solely on parent reports, which omit perspectives captured by other informants (e.g., second parents, teachers). Future research should incorporate multiple informants to validate or extend these findings. Additionally, checklist-based surveys may not fully capture internalizing problems, as gold-standard assessments typically require more in-depth interviews, given that such symptoms are often less observable. We also used only two of the three subscales of internalizing symptoms and excluded somatic complaints. Items were recoded as binary (0 vs. 1–2) due to low endorsement rates in these non-clinical samples; although this approach is common in similar contexts, it discards severity information, and its impact on factor structure has not been formally tested.

Furthermore, changes in symptoms across development may also be qualitative. That is, even when symptom intensity remains stable, their context, risk profile, and other characteristics can shift as children develop, which our study was not able to capture. The between-sex MI analysis in Sample B was limited to two factors due to identification issues with the third factor. This restricts comparability of the withdrawal dimension across sexes in that sample. Finally, to analyze sex differences, we used sex assigned at birth. However, gender identity can evolve over time, and our binary analytical framework could not capture the experiences of transgender or gender-diverse youth (McAdaragh et al., 2024). The interaction between non-binary identities and the dimensions we studied remains an open question.

Further validation is needed beyond the MI established here. Future research should examine convergent and discriminant validity by testing how these dimensions relate to clinical diagnoses, treatment outcomes, and established measures. It would also be valuable to clarify under what conditions social withdrawal differentiates into a distinct depressive dimension, whether the steep rise in girls’ Dep/With reflects new risk factors or continuity of earlier vulnerability, and whether boys’ decline in S/GA reflects genuine resolution or suppression of anxiety expression. Extending this framework to early childhood would help clarify whether these dimensions are detectable and psychometrically stable prior to age 7.

### Practical and Clinical Implications

The identification of psychometrically stable dimensions from around age 7 suggests that dimension-specific screening is feasible before adolescence. In particular, caregiver-reported self-worth concerns, such as feeling unloved, worthless, or inferior, may serve as early and relatively low-threshold indicators of depressive vulnerability, even at subclinical levels.

The distinct developmental trajectories of these dimensions also point to different intervention timing and targets. Interventions focusing on self-worth and self-critical thinking may be most effective if initiated early and sustained through adolescence. In contrast, interventions targeting interpersonal fear and concerns about social evaluation may be especially relevant for girls during the transition to adolescence. Clinicians should also be cautious of treating social withdrawal as benign, as disinterest in social situations may contribute to depressive symptoms during adolescence. A child presenting with depressed mood or loss of interest may be struggling with a sense of worthlessness, or alternatively with social fears and withdrawal — and these may call for different clinical responses.

Our findings also connect to the well-known comorbidity between depression and anxiety during adolescence, which appears to be especially strong in girls (Cohen et al., 2017; Kouros & Garber, 2014). One possible mechanism underlying this comorbidity involves social factors. In our analysis, the anxiety dimension was mainly defined by social-evaluative concerns, such as shyness, self-consciousness, and embarrassment. At the same time, social withdrawal and socially based self-worth were salient markers of the depressive dimensions. Thus, shared social and interpersonal vulnerabilities, such as heightened sensitivity to social evaluation, peer-related stress, and social withdrawal, may increase anxious and depressive symptoms together, which could help explain the parallel rise of these two distinguishable dimensions of anxiety and depression. This may occur especially in girls, who are often more reactive to interpersonal stress (Rudolph & Conley, 2005). Understanding these social mechanisms remains an important goal for future work and may improve interventions targeting the social pathway to depression (Nelemans et al., 2019).

## Conclusion

The present study provides evidence that internalizing symptoms in youth comprise multiple psychometrically stable dimensions rather than a single broad construct. Low self-worth/depression and social/general anxiety were reliable and stable from middle childhood to adolescence across both samples; depression/withdrawal emerged as a third dimension in Sample A. We showed that these dimensions follow different developmental trajectories depending on age and sex. As the field moves toward more dimensional approaches to psychopathology, this kind of longitudinal evidence becomes especially important — it is not sufficient to demonstrate that dimensions exist at a single time point; they must also show stability across development, invariance across sex, and be detectable in younger ages. Our findings provide empirical support for these models, pointing toward a developmentally informed approach to identifying and treating internalizing vulnerability in youth.

## Data Availability

The current study’s design and analysis were not pre-registered. Select de-identified data from the Early Growth and Development Study from the ECHO Program are available through NICHD’s Data and Specimen Hub (DASH; https://dash.nichd.nih.gov/). If further information is needed, please contact the corresponding author.
